# Levels of lncRNA GAS5 in Plasma of Patients with Severe Traumatic Brain Injury: Correlation with Systemic Inflammation and Early Outcome

**DOI:** 10.3390/jcm11123319

**Published:** 2022-06-09

**Authors:** Jin Lei, Xiao Zhang, Rui Tan, Yu Li, Kai Zhao, Hongquan Niu

**Affiliations:** 1 Department of Neurosurgery, Tongji Hospital, Tongji Medical College, Huazhong University of Science and Technology, Wuhan 430030, China; doctorjinlei@163.com (J.L.); doctorruitan@163.com (R.T.); hqniu@tjh.tjmu.edu.cn (H.N.); 2 Data Science Center, Clinical Development Division, CSPC Pharmaceutical Group Co., Ltd., Wuhan 430030, China; simabamboo@163.com

**Keywords:** traumatic brain injury, expression, long non-coding RNA, biomarker, clinical study

## Abstract

Scientific efforts continue to concentrate on elucidating the complex molecular mechanisms underlying traumatic brain injury (TBI), and recent reports suggest that epigenetic regulation including long non-coding RNA (lncRNA) is involved. The present study aimed to investigate the plasma concentration of a long non-coding RNA, named growth arrest-specific 5 (GAS5), in a group of 45 patients with severe TBI (sTBI), and to analyze the correlations of GAS5 with TBI onset, injury severity, systemic inflammation, and early outcome of the patients. It was found that plasma GAS5 levels were substantially increased in sTBI patients compared with the relative controls (*p* < 0.001). Further, significantly higher expression of plasma GAS5 was observed in patients with a Glasgow Coma Scale (GCS) score of less than five (*p* = 0.002) or unfavorable outcome at discharge (*p* < 0.001). Circulating GAS5 expression had a negative correlation with GCS score (r = −0.406, *p* = 0.006), and positive correlations with white blood cell count (r = 0.473, *p* = 0.001), neutrophil count (r = 0.502, *p* < 0.001), and neutrophil/lymphocyte ratio (NLR) (r = 0.398, *p* = 0.007). Univariate and multivariate logistic regression analyses revealed that GCS score (OR = 0.318, 95% CI 0.132–0.767, *p* = 0.011) and GAS5 (OR = 2.771, 95% CI 1.025–7.494, *p* = 0.045) were the two independent predictors for early outcome of patients. The receiver operating characteristic (ROC) curves showed good prognostic values of GCS score (AUC = 0.856, 95% CI: 0.719–0.943) and GAS5 expression (AUC = 0.798, 95% CI: 0.651–0.903). Importantly, the combined use of them can improve the prognostic ability of TBI with an AUC of 0.895 (95% CI: 0.767–0.966). Collectively, our study indicated that the levels of lncRNA GAS5 in circulation were elevated following severe TBI and correlated well with injury severity and inflammatory parameters. In addition, GAS5 as well as GCS scores may have the potential to predict the early outcome of TBI patients.

## 1. Introduction

Traumatic brain injury (TBI) is one of the leading causes of death and disability worldwide, which results in a huge burden on families and society [1]. The prognosis for this kind of disease has been dismal, especially for the most severe type, i.e., severe TBI (sTBI) [2]. Moreover, evidence from clinical trials and meta-analyses suggests that no intervention shows clear efficacy in improving the outcome for TBI patients [3]. One of the explanations was that the pathophysiological mechanisms underlying TBI have not been clearly understood yet, and epigenetic mechanisms have been recently suggested to play a role [4].

Long non-coding RNAs (lncRNA), a class of RNA transcripts more than 200 nucleotides in length that are involved in the modulation of gene expression, have received reasonable attention during the last decade. Although lacking capacity to encode proteins, they show greater expression specificity and regulatory ability compared with protein-coding genes [5]. Moreover, lncRNAs have specific expression profiles in brain tissue, which suggests their unique role in the development and disorders of the central nervous system (CNS) [6]. It has been shown that a wide range of lncRNAs were expressed aberrantly following CNS injuries, including TBI [7]. Growth arrest-specific 5 (GAS5) is a kind of lncRNA that is abundant in growth-arrested cells and regulates cell susceptibility to apoptotic and other growth-related signals [8]. It was initially identified as a tumor suppressor gene, and was recently found to be overexpressed in experimental models of neonatal hypoxic/ischemic brain injury, stroke, and spinal cord injury [9,10,11,12]. Additionally, a previous microarray study suggested that the expression of GAS5 was elevated in rat hippocampus tissue after TBI [13]. However, the expression and clinical significance of GAS5 in TBI patients have not been evaluated so far.

On this account, in the present study, we explored the levels of GAS5 in a group of TBI patients and evaluated the correlations of GAS5 expression with TBI onset, severity, and inflammatory parameters after injury. In addition, the prognostic value of GAS5 was also investigated.

## 2. Methods

### 2.1. Study Population

From December 2017 to November 2019, a total of 45 severe TBI patients were recruited from Tongji Hospital, Tongji Medical College, Huazhong University of Science and Technology. The criteria for study inclusion were as follows: (1) an acute TBI less than 24 h before admission; (2) a Glasgow Coma Scale (GCS) score of 3–8 after resuscitation; (3) abnormal head CT scan. The exclusion criteria were TBI accompanied with a spontaneous brain hemorrhage, stroke, spinal cord injury, tumor, or autoimmune disease. To establish the normal reference range of GAS5, 45 control subjects who were admitted for surgical removal of benign scalp lipoma and without a history of CNS injuries, infection, or malignancies were included. Age (51.1 ± 15.2 vs. 49.6 ± 10.53, *p* = 0.569) and gender (male/female: 33/12 vs. 30/15, *p* = 0.490) did not differ significantly between TBI patients and control subjects.

For all patients, the demographic, clinical, and laboratory data were obtained from clinical records. A dilated pupil was defined as larger than 5 mm in diameter and did not return to normal under the light. The GCS score at the time of admission was measured to assess the conscious state and injury severity; the Glasgow Outcome Scale (GOS) score at discharge was used to evaluate the early outcome of TBI patients. The GOS is a five-point scale used to evaluate the neurological outcome as follows: 1 indicates death, 2 indicates a vegetative state, 3 indicates severe disability, 4 indicates moderate disability, and 5 indicates good recovery. In the subsequent analyses, the outcome of patients was further dichotomized into unfavorable outcome (UO, GOS 1–3) versus favorable outcome (FO, GOS 4–5).

### 2.2. Sample Collection

Whole blood samples were collected on admission from all patients and control participants in ethylenediaminetetraacetic acid (EDTA)-coated tubes and were centrifuged (1000× *g* for 10 min at 4 °C) to obtain the plasma. Plasma was then transferred to RNase-free microcentrifuge tubes on ice and centrifuged at 3200× *g* for 15 min to remove platelets. After separation, plasma samples were flash frozen and stored at −80 °C until further analysis. The patient inclusion and sample collection procedure referred to the guidelines of the Ethics Committee of our hospital and written informed consent was obtained from all patients or their families/relatives.

### 2.3. RNA Extraction and Plasma GAS5 Assessment

Before RNA extraction, Caenorhabditis elegans cel-miR-39 (Sevicebio Technology, Wuhan, China), as an external reference, was added into the plasma samples at a final concentration of 0.2 nM. Then, total RNAs in plasma were isolated using Trizol reagent (Invitrogen, Carlsbad, CA, USA) according to the manufacturer’s instructions. The purity and concentration of total RNAs were determined by a NanoDrop spectrophotometer (Thermo Scientific, Waltham, MA, USA). RNA was reverse transcribed into cDNA with GAS5 gene-specific primers and cel-miR-39 RT primers by using Hifair III 1st Strand cDNA Synthesis Supermix (Yeasen Biotechnology, Shanghai, China) in a 20 μL system following the manufacturer’s protocol (25 °C for 5 min, 55 °C for 15 min and 85 °C for 5 min). Subsequently, quantitative real-time polymerase chain reaction (qRT-PCR) was performed using Hieff qPCR SYBR Green Master mix (Yeasen Biotechnology, Shanghai, China) with QuantStudio^TM^ 3 system (ThermoFisher Scientific, Waltham, MA, USA). The reaction was incubated at 95 °C for 5 min, followed by 40 cycles at 95 °C for 10 s and 60 °C for 30 s, and a final melt cure stage at 60 °C for 1 min and 95 °C for 1 s. Calculation of cycle threshold (Ct) was analyzed and the relative expression level of lncRNA GAS5 was calculated by the 2^−ΔΔCt^ method with cel-miR-39 as the reference. The primers were designed as follows: GAS5, forward (5′→3′): TGTGGCTCTGGATAGCACCTTA, reverse (5′→3′): GTCTAATGCCTGTGTGCCAATG; cel-miR-39-3p-RT (5′→3′): CTCAACTGGTGTCGTGGAGTCGGCAATTCAGTTGAG CAAGCTGA; cel-miR-39-3p-S (5′→3′): ACACTCCAGCTGGGTCACCGGGTGTAAATC; Universal Primer-A (5′→3′): TGGTGTCGTGGAGTCG.

### 2.4. Statistical Analysis

Continuous variables were expressed as means ± standard deviation (SD) or medians (interquartile range), and categorical variables as percentages. Comparisons were made by a Student’s *t*-test, Wilcoxon rank-sum test, chi-squared test, or Fisher’s exact test, if appropriate. Spearman correlation test was used to explore the correlation between GAS5 expression and clinical variables of the patients. Univariate and multivariate logistic regression analyses were used to identify the risk factors associated with patient outcome. Further, receiver operating characteristic (ROC) curves, area under the ROC curves (AUCs), and corresponding confidence intervals (CIs) were constructed to determine the efficiency of plasma GAS5 in predicting the outcome of patients. Statistical analyses and graph production were performed with GraphPad Prism version 7 and MedCalc version 12. All tests were two-sided, and *p* < 0.05 was considered to be statistically significant.

## 3. Results

### 3.1. Clinical Basic Data

A total of 45 severely brain-injured patients were included during the study period. Overall, the mean age of the studied subjects was 51.1 ± 15.2 years, and 73.3% (33/45) of them were male. Traffic accident and fall were the top two causes of injury, accounting for 86.7% of the patients. The mean GCS score was 6.5 ± 1.7, and nearly one-third (13/45, 28.9%) of the patients were admitted with at least one dilated pupil. Thirty-two of forty-five (71.1%) patients received at least one neurosurgical operation during the hospital period, and they stayed in hospital for a mean of 11.1 days. The 45 patients were then divided into an unfavorable outcome group (UO group, n = 24) and a favorable outcome group (FO group, n = 21) according to their GOS score at discharge. The demographic and clinical characteristics of the two groups of patients are summarized in Table 1. No significant differences were detected between the two groups in terms of age, gender, cause of injury, pupil reaction, neurosurgery, and neutrophil/lymphocyte ratio (NLR). The UO group presented a significantly lower GCS score (5.5 ± 1.7 vs. 7.6 ± 0.9, *p* < 0.001) and a shorter length of hospital stay (8.7 ± 8.9 vs. 13.8 ± 7.6, *p* = 0.048) than those of the FO group. The white blood cell count and neutrophil count in the UO group were higher than those in the FO group (*p* < 0.05 for both comparisons).

### 3.2. Expression of GAS5 in Severe TBI Patients

**Figure 1:**
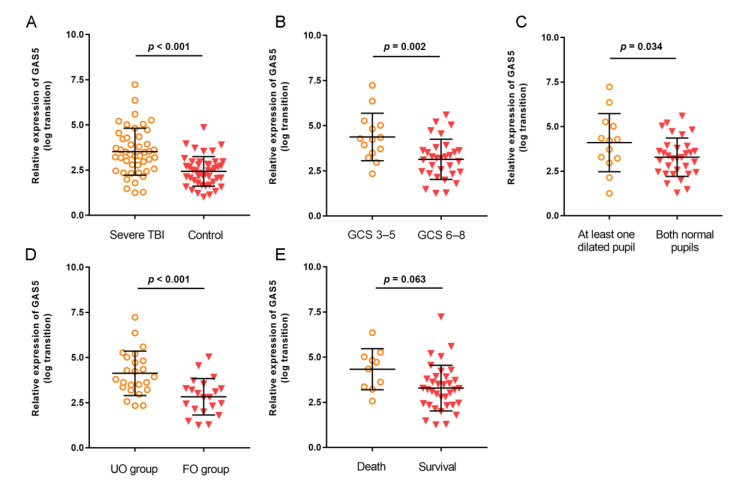
The relative levels of GAS5 in plasma samples of severe TBI patients and controls categorized by clinical variables: (**A**) severe TBI versus control; (**B**) GCS 3–5 versus GCS 6–8; (**C**) at least one dilated pupil versus both normal pupils; (**D**) unfavorable outcome (UO) group versus favorable outcome (FO) group; (**E**) death versus survival. Expression of GAS5 was expressed relative to its respective level of cel-miR-39. The bar represents mean and standard deviation.

To test GAS5 expression after severe TBI, blood samples from patients and their matched controls were collected and examined with a qRT-PCR test. The results showed that the mean level of GAS5 in sTBI patients was significantly higher than that in the controls (*p* < 0.001, Figure 1A). When categorized by GCS score, a significantly more elevated level of GAS5 in those patients with GCS 3–5 was observed than that in the patients with GCS 6–8 (*p* = 0.002, Figure 1B). For patients with at least one dilated pupil at admission, the mean GAS5 levels were higher than those with both normal pupils (*p* = 0.034, Figure 1C). Furthermore, for patients with unfavorable outcome, the GAS5 expression was much higher than those with favorable outcome (*p* < 0.001, Figure 1D). Moreover, the patients who died in the hospital showed a tendency towards higher GAS5 expression compared with survivors, although statistical significance was not reached (*p* = 0.063, Figure 1E).

### 3.3. Correlations of GAS5 Expression with Clinical and Inflammatory Parameters

**Figure 2:**
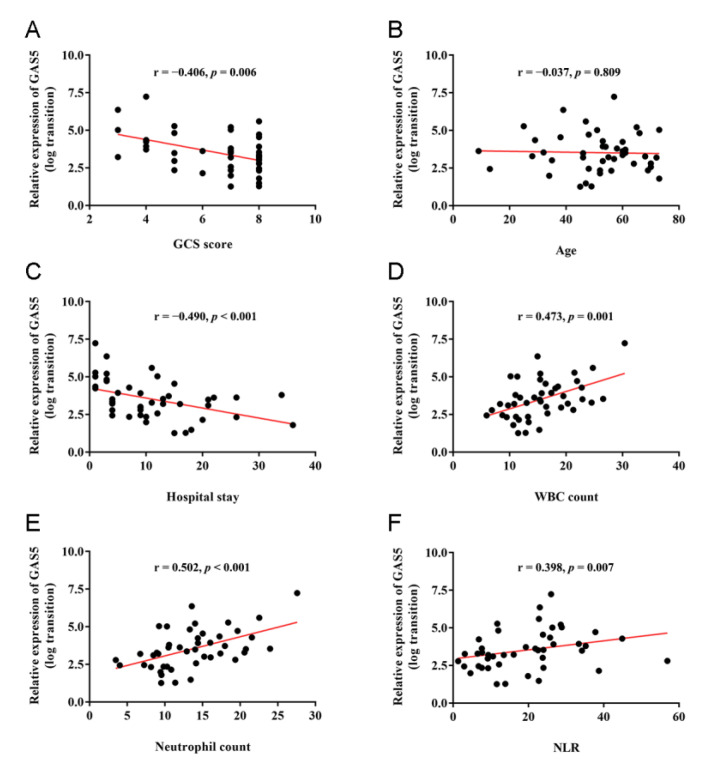
Correlations of plasma GAS5 expression with clinical and inflammatory parameters in TBI patients: (**A**) GAS5 versus GCS score; (**B**) GAS5 versus age; (**C**) GAS5 versus the length of hospital stay; (**D**) GAS5 versus white blood cell count; (**E**) GAS5 versus neutrophil count; (**F**) GAS5 and neutrophil/lymphocyte ratio. Spearman correlation coefficient (r) and *p* values are listed above each chart.

The correlations between plasma GAS5 expression and various variables of TBI patients were analyzed using Spearman correlation test. As shown in Figure 2, the plasma GAS5 level correlated significantly with GCS score (r = −0.406, *p* = 0.006), which is consistent with our initial data that the most severe TBI patients (GCS score 3–5) presented a higher GAS5 level. Further, no significant correlation between GAS5 and age (r = −0.037, *p* = 0.809), and a significantly negative correlation between GAS5 and the length of hospital stay (r = −0.490, *p* < 0.001), were observed. The correlation analysis also demonstrated that GAS5 expression correlated positively with several inflammatory parameters, including WBC count (r = 0.473, *p* = 0.001), neutrophil count (r = 0.502, *p* < 0.001), and NLR (r = 0.398, *p* = 0.007).

### 3.4. Prognostic Value of GAS5 Expression in Predicting Patient Outcome of Severe TBI

To determine the predictive ability of GAS5 expression for the outcome of severe TBI patients, we performed univariate and multivariate logistic regression analyses. The univariate analysis showed that GCS score, GAS5 expression, WBC count, and neutrophil count are potential risk factors for unfavorable outcome of patients. After incorporation into a multivariate logistic model, we found that GCS score (OR = 0.318, 95% CI 0.132–0.767, *p* = 0.011) and GAS5 expression (OR = 2.771, 95% CI 1.025–7.494, *p* = 0.045) were the two variables independently predictive of severe TBI patient outcome with statistical significance (Table 2).

**Figure 3:**
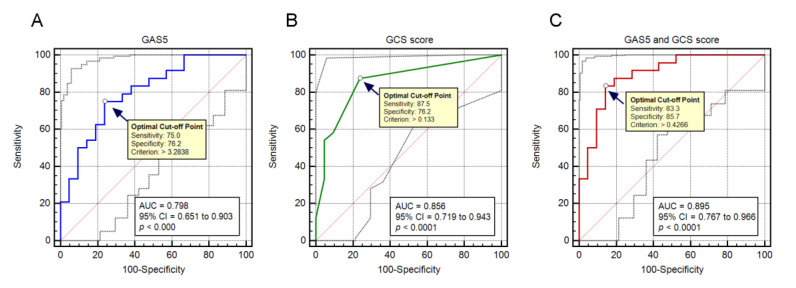
Receiver operating characteristic (ROC) curves of GAS5 (**A**), GCS score (**B**), and the combination of both (**C**), relative to early outcome of TBI patients.

The ROC analysis results showed that GAS5 level and GCS score reliably predicted the outcome of patients, with an AUC of 0.798 (95% CI: 0.651–0.903, *p* < 0.0001) and 0.856 (95% CI: 0.719–0.943, *p* < 0.0001), respectively. A GAS5 threshold value of 3.2838 was chosen and it showed that plasma GAS5 had a sensitivity of 75% and specificity of 76.2% for the prediction of unfavorable outcomes. The optimal cutoff point of the GCS score was 7.5 (prediction sensitivity: 87.5%; specificity: 76.2%). The combination of GAS5 expression and GCS score improved the prognostic ability with an AUC of 0.895 (95% CI: 0.767–0.966, *p* < 0.0001) (Figure 3).

## 4. Discussion

The present study showed that the plasma GAS5 levels of sTBI patients were significantly higher than those of controls. Moreover, the elevated expression of GAS5 was significantly correlated with injury severity, WBC and neutrophil counts, and NLR in the blood samples. In addition, GAS5 expression was an independent risk factor for the early outcome of patients with severe TBI and could predict the outcome with an AUC of 0.798. The combined use of GCS score and GAS5 expression significantly improved the prognostic prediction.

TBI has been one of the highest unmet needs within the field of brain research in recent decades. It is the most common cause of death and disability for people up to 40 years of age, and the incidence of TBI is still rising sharply worldwide due to increasing industrialization in low- and middle-income countries [14]. However, the outcome for patients with TBI, especially severe TBI, was generally poor, despite the improved surgical techniques and neurocritical care [15]. Moreover, few of the current interventions proved to be efficacious when evaluated by hundreds of trials. Since the mechanism of TBI is extremely complicated, which is characterized by an initial primary injury and a later secondary injury, including hypoxic–ischemic brain damage, inflammation, blood–brain barrier (BBB) disruption, and oxidative stress, the exploration of key molecules that are involved in the pathology of brain injury would be of particular importance [16].

A growing number of lncRNAs have recently been found to be involved in the pathophysiological processes of brain injury, for instance: apoptosis, autophagy, inflammation, BBB stabilization, and angiogenesis [7]. A previous study has reported that GAS5 promoted microglial M1 polarization and suppressed M2 polarization via the inhibition of TRF4 transcription in a mice model of experimental autoimmune encephalomyelitis [17]. In addition, some data indicated that GAS5 was increased in the rat hippocampus following TBI and exhibited a pro-apoptotic effect by the miR-335/Rasa1 pathway [18]. Furthermore, highly expressed GAS5 was observed in the injured brain tissue of middle cerebral artery occlusion (MCAO) and hypoxia/ischemic brain damage (HIBD), and mediates neuronal death in ischemic brain injury [10,11]. Our study would appear to be an important supplement to the above-mentioned studies, contributes to a better understanding of the pathologies and the role of GAS5 after brain injury, and facilitates the development of novel therapeutic options for these kinds of diseases.

Apart from the involvement in CNS injuries, GAS5 has also been studied as an inflammation-related gene, due to its direct influence on glucocorticoid receptor (GR) function, which is considered as a repressor of GR action [19]. Altered expression of lncRNA GAS5 in blood and significant correlation with systemic inflammation have been reported in patients with rheumatoid arthritis, systemic lupus erythematosus, mycoplasma pneumoniae pneumonia, and sepsis, highlighting the role of GAS5 in acute and chronic inflammatory diseases [20,21,22,23]. Systemic inflammation is an important hallmark of TBI, which is associated with the poor outcome of patients [24,25]. In our study, the levels of GAS5 in circulation were significantly increased in patients with sTBI, and correlated with WBC and neutrophil counts and NLR, indicating that GAS5 might be involved in inflammatory processes of TBI. However, the origin of GAS5 and its precise role in post-injury inflammation are still unknown. Whether the elevated GAS5 was leaked from injured brain tissue via disrupted BBB or just existed in blood as an indication of systemic inflammation still needs further exploration.

In the present study, we found GCS score and GAS5 expression as the two independent predictors for unfavorable outcomes of severely brain-injured patients, with an AUC of 0.856 and 0.798, respectively. GCS score is a clinical assessment tool that has been reported to be a good predictor for injury severity and progression of brain damage [26]. However, it has been recently criticized for being subjective and susceptible to complex clinical situations, such as sedation or shock [27]. Therefore, the identification of valid biomarkers that can provide objective and quantitative endpoints for injury evaluation and outcome prediction is of particular importance, and this kind of research is currently ongoing. We found, in the current study, that GAS5 can provide moderate ability (AUC: 0.798) in predicting patient outcome with good sensitivity and specificity. Moreover, the combination of GAS5 expression and GCS score could reach an AUC of 0.895. These findings suggested that lncRNAs may act as potentially useful biomarkers of brain injury and should be considered in future investigations.

There are several limitations in our study that need to be cautiously interpreted. Firstly, the sample size was small due to the slow recruitment of eligible patients during the research period. However, the study was much more preliminary than validatory in nature, with some significant results observed. Thus, we expect a study with a larger sample size to confirm and extend our findings. Secondly, stability is a crucial prerequisite for any circulating biomarker [28]. We did not evaluate the stability in plasma under harsh conditions, such as prolonged exposure of the blood samples at room temperature, or repeated freeze–thaw cycles. Thirdly, we used WBC and neutrophil counts and NLR as indicators for systemic inflammation of the patients, since they are established inflammatory markers following TBI, as supported by previous reports [29,30]. If possible, more inflammatory markers, such as C-reactive protein (CRP), matrix metalloproteinases (MMPs), or pro-inflammatory cytokines, are suggested in future studies to systematically evaluate the inflammatory response in circulation of TBI patients.

In conclusion, this study explored the expression of lncRNA GAS5 in severe TBI, and its correlations with injury severity, inflammatory response, and prognosis of the patients. The expression of GAS5 in plasma may be used as a potential new biomarker in the diagnosis and outcome prediction of TBI. Owing to the preliminary study design, our findings still need further validation; meanwhile, the specific mechanism of GAS5 in TBI needs further investigation.

## Figures and Tables

**Table 1:** Demographic and clinical characteristics of the severe TBI patients grouped by GOS score at discharge.

	UO Group (n = 24)	FO Group (n = 21)	Total (n = 45)	*p* Value
Age (years)	52.4 ± 14.9	49.7 ± 15.9	51.1 ± 15.2	0.552
Gender (Male/Female)	20/4	13/8	33/12	0.105
Cause of injury, n (%)				0.904
Traffic accident	11 (45.8%)	12 (57.1%)	23 (51.1%)	
Fall	10 (41.7%)	6 (28.6%)	16 (35.6%)	
Assault	2 (8.3%)	2 (9.5%)	4 (8.9%)	
Other	1 (4.2%)	1 (4.8%)	2 (4.4%)	
GCS score (point)	5.5 ± 1.7	7.6 ± 0.9	6.5 ± 1.7	<0.001
Pupil reaction, n (%)				
Both normal	15 (62.5%)	17 (81.0%)	32 (71.1%)	
One pupil dilated	5 (20.8%)	4 (19.0%)	9 (20%)	0.122
Both pupils dilated	4 (16.7%)	0 (0%)	4 (8.9%)	
Neurosurgery	19 (79.1%)	13 (61.9%)	32 (71.1%)	0.203
Length of hospital stay (days)	8.7 ± 8.9	13.8 ± 7.6	11.1 ± 8.7	0.048
WBC (10^3^/μL)	17.2 ± 5.1	13.8 ± 5.5	15.6 ± 5.5	0.040
Neutrophil count (10^3^/μL)	15.2 ± 4.8	11.9 ± 5.3	13.6 ± 5.3	0.037
NLR	21.3 ± 10.9	17.4 ± 13.6	19.4 ± 12.2	0.296

Abbreviations: UO, unfavorable outcome; FO, favorable outcome; GOS, Glasgow Outcome Scale; GCS, Glasgow Coma Scale; WBC, white blood cell; NLR, neutrophil/lymphocyte ratio.

**Table 2:** Univariate and multivariate logistic analyses for the prognostic factors of severe TBI.

Variables	Univariate Analysis			Multivariate Analysis		
	OR	95% CI	*p*-Value	OR	95% CI	*p*-Value
GCS score	0.336	0.169–0.666	0.002	0.318	0.132–0.767	0.011
GAS5 expression	3.119	1.462–6.652	0.003	2.771	1.025–7.494	0.045
Age	1.012	0.973–1.053	0.543			
Sex	0.325	0.081–1.303	0.113			
Cause of injury						
Pupil reaction	0.392	0.100–1.539	0.180			
Neurosurgery	2.338	0.624–8.766	0.208			
Lohs	0.927	0.857–1.004	0.161			
WBC count	1.134	1.001–1.285	0.049	1.547	0.624–3.836	0.347
Neutrophil count	1.144	1.003–1.306	0.045	1.146	0.246–1.697	0.376
NLR	1.028	0.977–1.082	0.285			

Abbreviations: OR, odds ratio; CI, confidence interval; Lohs, length of hospital stay; NLR, neutrophil/lymphocyte ratio.

## Data Availability

All data generated during this study are included in this article.
